# Risque d’émergence de maladies arbovirales transmises par les moustiques au Maroc: revue bibliographique

**DOI:** 10.48327/mtsi.v5i4.2025.671

**Published:** 2025-08-21

**Authors:** Samya JDIAA, Hanan HAZYOUN, Moulay Anass LOUAH, Oumnia HIMMI

**Affiliations:** 1 LESCB URL/CNRST N°18, FS, Université Abdelmalek Essaâdi, Tétouan, Maroc; 2 Laboratoire de Géo-biodiversité et patrimoine naturel (GEOBIOL). Institut scientifique, Uni-versité Mohammed V de Rabat. Avenue Ibn Battota. BP. 703. Rabat-Agdal, Maroc

**Keywords:** Maladies à transmission vectorielle, Arbovirus, Culicidae, Risques d’émergence, Maroc, Afrique du Nord, Vector-borne diseases, Arboviruses, Culicidae, Risk of emergence, Morocco, North Africa

## Abstract

**Introduction:**

L’ émergence des arbovirus (virus transmis par les arthropodes) figure aujourd’hui parmi les plus grands problèmes de santé au monde. La mondialisation des échanges et des voyages ainsi que l’urbanisation sauvage de nombreuses villes ont créé des conditions propices à l’établissement de moustiques vecteurs offrant des possibilités d’introduction d’arbovirus.

**Méthode:**

Dans cette revue, nous résumons les données historiques et récentes sur la situation des maladies transmises par les arthropodes et leurs vecteurs au Maroc.

**Résultats:**

Il existe un risque d’émergence et d’épidémies au Maroc en raison de la circulation récente du virus du Nil occidental parmi les équidés et les oiseaux selon les études séroépidémiologiques, ainsi que dans les populations de moustiques *Culex.* L’introduction de nouvelles espèces de moustiques invasives telles que *Aedes albopictus,* vecteur de la dengue, du Zika et du chikungunya, exacerbe encore ce risque. Une quinzaine despèces de moustiques, vecteurs de transmission d’agents pathogènes pour les humains et les animaux, ont été signalées au Maroc.

**Conclusion:**

La compréhension des épidémies d’arboviroses et de la transmission des virus nécessite une étude préalable des moustiques vecteurs, pour une appréhension globale du risque actuel auquel notre pays est confronté et pour une bonne préparation face aux menaces futures.

## Abstract (en)

**Introduction:**

The emergence of arboviruses, or viruses transmitted by arthropods, is now one of the world’s biggest health problems. The globalization of trade and travel, coupled with uncontrolled urbanization in many cities, has created ideal conditions for mosquito vectors to establish themselves and introduced opportunities for arboviruses to spread.

**Method:**

In this review, we summarize historical and recent data on arthropod-borne diseases and their vectors in Morocco.

**Results:**

According to seroepidemiological studies there is a risk of emergence and epidemics in Morocco due to the recent circulation of West Nile virus among equids and birds as well as in populations of *Culex* mosquitoes. The introduction of new invasive mosquito species, such as *Aedes albopictus* (a vector of dengue, Zika, and chikungunya), further exacerbates this risk. About fifteen mosquito species as vectors for transmitting pathogens to humans and animals have been reported in Morocco.

**Conclusion:**

In order to understand arbovirus epidemics and virus transmission, it is necessary to study mosquito vectors first, in order to gain a comprehensive awareness of the current risk facing our country and to be well prepared for future threats.

## Introduction

Les moustiques ont une répartition mondiale et sont les vecteurs de plusieurs arbovirus (virus transmis par les arthropodes) qui infectent les humains et les animaux [2]. Les principaux virus transmis par les moustiques appartiennent à trois familles: Flaviviridae (virus du Nil occidental, virus de la dengue, virus de la fièvre jaune), Togaviridae (virus du chikungunya, virus O’nyong-Nyong) et Phenuiviridae (virus de la fièvre de la vallée du Rift) [1,2].

Plus de 700 millions d’infections annuelles dans le monde et près d’un million de décès sont directement liés aux maladies à transmission vectorielle [68,97]. En Afrique, ces infections représentent un risque majeur: 271 millions de personnes (23% de la population) sont exposées au chikungunya, 750 millions (63%) à la dengue, 21 millions (2%) à la fièvre jaune et 406 millions (34%) au virus Zika. Au total, 831 millions d’Africains (70%) vivent dans des zones à risque pour au moins l’une de ces maladies [95].

Le virus du Nil occidental (VNO) dont la présence a été évoquée dès 1982 a été isolé pour la première fois au Maroc en 1996 et a été responsable de trois épizooties équines [35]. En 2008, une enquête sérologique sur les oiseaux sauvages a confirmé la circulation du VNO au Maroc [43] et en 2012, la première infection humaine par le VNO a été documentée par des preuves sérologiques [37]. Plus récemment, d’autres arbovirus tels que les virus de la dengue et du chikungunya ont été identifiés chez des patients marocains ayant séjourné respectivement en Côte d’Ivoire et au Bangladesh [10,11].

**Figure 1:**
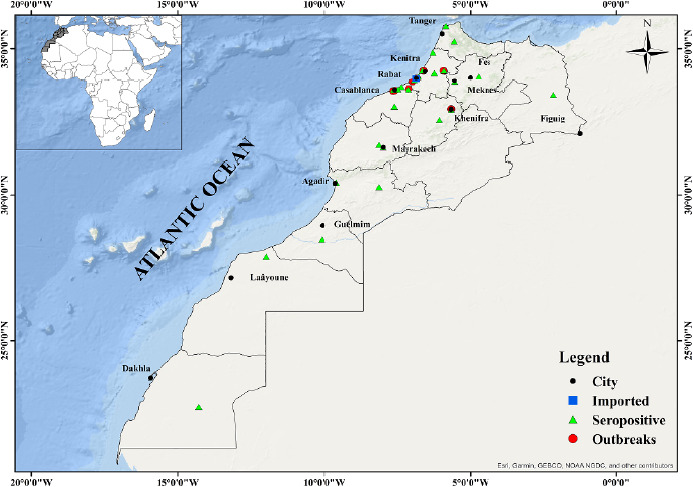
Distribution géographique des arbovirus (cas importés, hôtes séropositifs, foyers) par région au Maroc depuis leur apparition

Compte tenu de sa situation géographique, de son climat et de son exposition à la mondialisation des échanges et des voyages (notamment avec l’Europe et l’Afrique subsaharienne), le Maroc constitue une zone à haut risque pour l’émergence ou la réémergence d’agents pathogènes (Fig. 1). Cette situation favorise également la propagation rapide des insectes vecteurs, exposant ainsi une grande partie de la population à ces menaces sanitaires [45,73].

Cette étude vise à identifier les principaux arbovirus d’importance pour la santé humaine au Maroc et leurs vecteurs épidémiques, ainsi que les conditions favorisant leur émergence.

## Matériel et méthodes

Les articles pertinents ont été recherchés, sélectionnés et inclus conformément aux directives du guide PRISMA *(Preferred Reporting Items for Systematic Reviews and Meta-Analyses)* [61,72]. Les études publiées ont été recherchées depuis l’identification des virus au Maroc jusqu’à aujourd’hui. Elles sont basées sur des recherches bibliographiques systématiques. Celles-ci incluent la base de données Culicidae of Morocco [89], qui retrace l’histoire des moustiques dans le pays de 1916 à 2017 et différents moteurs de recherche (Web of Science, Google Scholar, Scopus, Science direct, PubMed, et Organisation mondiale de la santé). Les mots-clés suivants ont été utilisés: les termes de recherche comprenaient « Maroc » et « Mosquito-borne virus, mosquito-borne diseases, MBV », ou « arbovirus »> ou « West Nile virus, WNV », ou « Dengue virus, DENV », ou « Rift Valley fever virus, RVFV »>, ou « Chikungunya virus, CHIKV ». Ensuite, pour « largir encore le champ de notre recherche, nous avons utilis » les termes « vector-borne disease genus », « Africa » and « emergence ».

**Figure 2:**
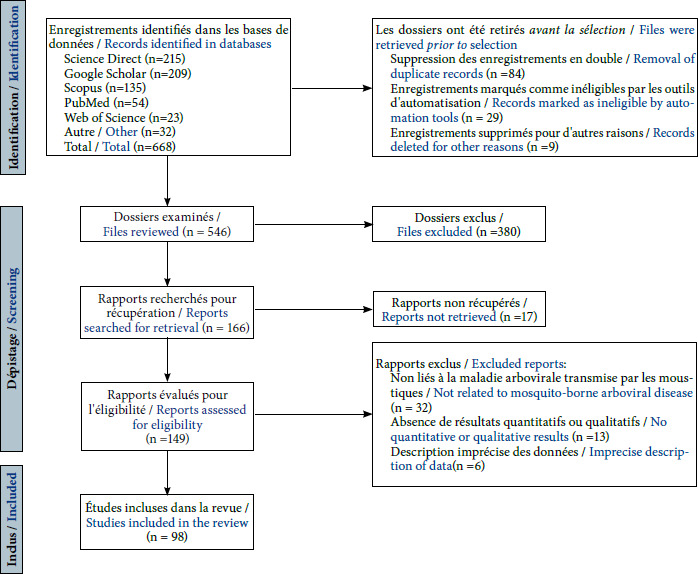
Identification des études par le biais de bases de données et de registres

Afin de garantir l’inclusion des études les plus pertinentes, une série de critères a été utilisée (études de recherche originales, études se référant au genre des maladies transmises par vecteurs) pour les documents de conférence et les articles de journaux concernant les arbovirus transmis par les moustiques. Les études qui ne répondaient pas aux critères d’inclusion (par exemple, les doublons, l’absence d’accès au texte intégral ou la langue autre que français ou l’anglais ont été exclues. La Figure 2 donne un aperçu détaillé du processus de recherche, en soulignant les critères d’inclusion et d’exclusion utilisés dans la méthodologie de sélection des publications.

## Résultats

Au total, 668 articles ont été extraits des bases de données. Après application des critères d’exclusion, 208 articles ont été retenus pour une analyse plus approfondie. Parmi ceux-ci, 52 ont été exclus car ils ne remplissaient pas les critères d’inclusion. Enfin, 97 articles portant sur l’émergence des maladies arbovirales transmises par les moustiques en tant que vecteurs ont été identifiés. L’organigramme PRISMA illustre la méthodologie employée pour la recherche et la sélection des études relatives aux maladies arbovirales transmises par les moustiques [61,72]).

### Aperçu historique des arbovirus et de leur épidémiologie actuelle au Maroc, en mettant l’accent sur leur distribution et leur vecteur

Les arbovirus représentent un problème de santé important à l’échelle mondiale et pourraient devenir un problème de santé publique important au Maroc.

Au total, 43 espèces de moustiques ont été signalées dans ce pays [91]. Parmi elles, 14 Culicinae ont été confirmées comme vecteurs potentiels pour la transmission d’agents pathogènes aux humains et/ou aux animaux [90]. Le Tableau I présente un résumé des principaux arbovirus et de leurs vecteurs identifiés entre 1996 et 2023.

**Tableau I:** Aperçu historique des arbovirus et de leur situation épidémiologique actuelle au Maroc, en mettant l’accent sur leur distribution et leurs vecteurs

Famille	Genre	Nom de la maladie	Hôtes amplificateurs	Année	Histoire au Maroc	Spécimen	Localités	Vecteur principal	Références
Phenuiviridae	*Phlebovirus*	Fièvre de la vallée du Rift (FVR)	Bovins, ovins, chameaux		Au Maroc, la FVR n’a pas été enregistrée, mais le virus est toujours présent aux frontières du pays avec la Mauritanie			*Cx. pipiens, Ae. vexans*	7,21,44,52,56,89
Flaviviridae	*Orthoflavivirus,*	Virus du Nil occidental (VNO)	Oiseaux	1996	Le premier rapport sur le VNO	Chevaux, humains	La zone côtière atlantique du nord-ouest du Maroc comprend les villes suivantes: Rabat, Kénitra, Khénifra, Benslimane et Sidi Slimane, ainsi que Casablanca	*Cx. pipiens, Ae. caspius, Ae. detritus, Ae. vexans, Ae. aegypti, Cx. modestus, Cx. mimeticus, Cx. perexiguus, Cx. theileri, Cx. impudicus, Cq. richiardii, Or. pulcripalpis*	6,8, 9,18,32,35,37,38,39,43,48,52,83,89
				2003	Réémergence du VNO	Chevaux	Kenitra, Khenifra, Benslimane, Sidi Slimane, Casablanca		32,83
				2008	Circulation du VNO au Maroc	Oiseaux sauvages	Province		43
				2010	Réémergence du VNO	Chevaux	Régions		32,37
				2012	Un test sérologique	Humains (cas humains confirmés), Le VNO chez 11,8% de 499 personnes en bonne santé	Régions		37
				2016	Séroprévalence	Chevaux et chiens	Agadir, Benslimane, Casablanca, Kenitra, Khenifra, Marrakech, Meknès, Salé, Sidi Slimane, Temara		32
Flaviviridae	*Orthoflavivirus*	Virus du Nil occidental (VNO)	Oiseaux	2017	Séroprévalence	Chevaux, le VNO chez 31% des 840 chevaux	Plateaux atlantiques du Gharb et de la région pré-Rif, Région des plaines et plateaux du Nord Atlas, Montagnes de l’Atlas et région pré-Atlas, Plaines et plateaux de la région orientale	*Cx. pipiens, Ae. caspius, Ae. detritus, Ae. vexans, Ae. aegypti, Cx. modestus, Cx. mimeticus, Cx. perexiguus, Cx. theileri, Cx. impudicus, Cq. richiardii, Or. pulcripalp*	16
				2020	Séroprévalence	Moustiques, Chevaux	Mohammedia & Casablanca, Moulay, Tanger, Benslimane		9
				2021	Séroprévalence	Humains, Oiseaux (oiseaux sauvages?)	Casablanca		8
				2021	Séroprévalence	Chevaux	Tanger, Tétouan, Al Hoceima L’Oriental Fès, Meknès, Beni Mellal, Khénifra, Rabat, Salé, Kénitra, Casablanca, Settat, Marrakech, Safi, Souss, Massa, Guelmim, Oued Noun, Laâyoune, Sakia El Hamra Dakhla, Oued Ed Dahab, Drâa, Tafilalet (la petite population de chevaux dans cette région)		48
		Usutu (USUV)	Oiseaux servant d’hôtes amplificateurs	2016	Enquête sérologique	Chevaux, Chiens militaires	Agadir, Benslimane, Casablanca, Kenitra, Khenifra, Marrakech, Meknès, Salé, Sidi Slimane, Temara	*Cx. pipiens, Ae. albopictus, Ae. caspius*	3,22,29,32,78
		Dengue (DENV)	Primates, humains	2018	Deux cas d’importation de la dengue au Maroc	Humains (deux patients originaires de Côte d’Ivoire, un Marocain et un Ivoirien qui ont séjourné à Abidjan pendant la période de l’épidémie de 2017)	Au Centre de virologie, maladies infectieuses et tropicales (CVITD) de l’Hôpital militaire Mohamed V à Rabat-Maroc	*Ae. albopictus Ae. aegypti*	10,11,17,64,89
Flaviviridae	*Orthoflavivirus*	Zika (ZIKV)	Primates, humains		Aucun cas dû au ZIKV n’a été signalé dans les pays de la région de la Méditerranée occidentale			*Ae. albopictus, Ae. aegypti*	41,49,52,67,79,90
		Fièvre jaune (YFV)	Singes en Afrique subsaharienne, primates non humains en tant qu’hôtes réservoirs		Aucun cas de YFV au Maroc			*Ae. aegypti, Ae. albopictus*	6,33,71,80
Idem Togaviridae	*Alphavirus*	Chikungunya (CHIKV)	Primates, humains	2017	Premier cas importé de Chikungunya au Maroc et en Afrique du Nord	La patiente est retournée au Maroc le 15 août 2017, après avoir séjourné à Dhaka-Bangladesh pendant 18 mois	Au Centre de virologie, maladies infectieuses et tropicales (CVITD) de l’Hôpital militaire Mohamed V à Rabat-Maroc	*Ae. albopictus, Ae. detritus, Ae. aegypti*	11,17,41,49,67,79,90

### Virus du Nil occidental (VNO)

Le VNO est un virus de la famille des Flaviviridae (genre *Orthoflavivirus)* transmis par plusieurs genres de moustiques. Il s’agit d’un arbovirus zoonotique maintenu dans la nature dans un cycle de transmission enzootique entre les oiseaux, principalement des passereaux, et les moustiques ornithophiles du genre *Culex.* Les oiseaux contribuent à la propagation du virus et agissent comme des hôtes amplificateurs. Le VNO peut également infecter les mammifères, y compris les humains, mais ces derniers sont considérés comme des hôtes « sans issue », c’est-à-dire que même s’ils sont infectés, le niveau de virémie qu’ils produisent est insuffisant pour infecter les moustiques et donc transmettre la maladie [20].

Les infections par le VNO chez l’humain sont généralement asymptomatiques (environ 80%) [74]. Une infection déclarée peut se présenter sous la forme d’un syndrome grippal (fièvre du Nil occidental), souvent accompagné d’une éruption cutanée ou, plus rarement, sous la forme d’un syndrome neuro-invasif avec méningoencéphalite, paralysie flasque dont le taux de létalité est inférieur à 10% (maladie neuro-invasive du Nil occidental) [23]. Environ 1% des personnes atteintes développent des symptômes graves [74]. Le VNO a été découvert pour la première fois en 1937 dans le sang d’une femme originaire de la province ougandaise du Nil occidental qui souffrait d’une légère maladie fébrile [86]. Depuis lors, des cas sporadiques et des flambées importantes de fièvre du Nil occidental ont été signalés en Afrique, dans certaines parties de l’Europe, au Moyen-Orient, en Asie occidentale, en Australie et dans les Amériques pour la première fois en 1999 au nord puis extension au centre et au sud) [13,46,63]. Des foyers particulièrement importants ont été documentés en Grèce, en Palestine, en Roumanie, en Russie, en France et aux États-Unis [97].

Au Maroc (Tableau II), selon les informations officielles des Services vétérinaires nationaux, la maladie à VNO est apparue pour la première fois en 1996, lorsqu’une épizootie a entraîné la mort de 42 chevaux (sur 94 cas) et d’un humain [43,88]. Par la suite, en 2003, la maladie s’est déclarée chez neuf chevaux [83]. En 2010, une réapparition du virus a été observée dans le centre et le nord-ouest du pays (Mohammedia, Casablanca, Benslimane, Khemisset), avec 17 cas équins confirmés parmi 111 cas suspects, entraînant 8 décès [16]. Depuis lors, aucun cas clinique de la maladie n’a été signalé dans le pays.

**Tableau II:** Séroprévalence du Virus du Nil Occidental (VNO) au Maroc

Origine géographique	Population étudiée	Échantillons	Positivité	Référence
Sidi Allai Tazi, Sidi Kacem Province	Oiseaux sauvages	346	4%	43
Meknes, Rabat, Kenitra	Personnes saines	499	11,8%	37
Kenitra, Khenifra, Benslimane, Sidi Slimane, Casablanca	Chevaux militaires	49	60%	32
	Chiens militaires	231	62%	32
Plateaux atlantiques du Gharb, Plaines et plateaux de l’Atlas du Nord, Montagnes de l’Atlas et région pré-Atlas, plaines et plateaux de l’Oriental	Chevaux	840	31%	16
Mohammedia, Moulay Bouselham, Tanger	Chevaux	92	33,7%	9
11 (sur 12) régions du pays	Chevaux	1 171	21,8%	48

Au total, sept études séro-épidémiologiques ont été menées dans la région du Nord. Une étude a également été conduite dans 11 des 12 régions du Maroc, montrant une circulation du VNO chez 4% de 346 oiseaux sauvages indigènes testés [43], 11,8% de 499 personnes en bonne santé [37], 60% et 62% des 49 chevaux et 231 chiens militaires) testés [32]. Trois enquêtes sur des chevaux ont donné 31% positifs sur 840 [16], 33,7% sur 92 [9] et 21,8% (255 sur 1 171) [48].

De même, une étude entomologique a confirmé la circulation du virus parmi les populations de moustiques *Culex* dans le pays [9]. Une deuxième étude récente sur la surveillance des moustiques a confirmé la présence du virus dans les populations de *Culex* du pays [9]. Cinquante-six moustiques supplémentaires (56 *Cx. pipiens pipiens)* de la région de Marrakech-Safi ont été testés positifs au VNO. C’est la première fois que la circulation de ce virus est documentée chez les moustiques *Cx. pipiens* dans le centre du Maroc [70].

### Virus de la fièvre de la vallée du Rift (VFVR)

Le VFVR est un arbovirus zoonotique qui affecte principalement les bovins, les moutons, les chèvres et les chameaux mais aussi les humains. Le VFVR appartient à la famille des Phenuiviridae, genre *Phlebovirus* [58], et a été identifié pour la première fois au Kenya en 1931 [57]. Il a été responsable de nombreuses épidémies et épizooties en Afrique, notamment en Afrique du Nord-Ouest (Mauritanie) et en Afrique de l’Ouest (Sénégal). La circulation du VFVR est restée limitée à l’Afrique jusqu’en 2000, date à laquelle les premiers cas hors du continent africain ont été rapportés en Arabie Saoudite et au Yémen [58].

Le VFVR n’a jamais été signalé au Maroc [7]. Cependant, le virus circule toujours le long des frontières communes avec la Mauritanie.

En 2010 en Mauritanie, 30 cas humains et 26 cas animaux (chèvres, moutons et dromadaires) ont été rapportés et le rôle du dromadaire *(Camelus dromedarius)* dans l’amplification locale du virus a été suggéré pour la première fois [36,42]. En 2015 puis en 2020,31 puis 78 cas humains de FVR ont été confirmés [12,19]. Sept espèces de moustiques connues pour être des vecteurs du VFVR et appartenant à trois genres (*Culex* spp., *Aedes* spp., *Mansonia* spp.), ont été détectées en Mauritanie [12].

Le Maroc a été identifié par l’OMS comme étant à risque d’émergence de la FVR en raison de sa frontière commune avec la Mauritanie. Le potentiel de propagation du virus de la Mauritanie au Maroc, puis à l’Europe, a été reconnu dans un autre rapport de l’Autorité européenne de sécurité des aliments en 2020 [65].

### Dengue (DENV)

La dengue est due au DENV (famille des Flaviviridae, genre *Orthoflavivirus).* Des épidémies de dengue ont été documentées en Afrique depuis le 19^e^ siècle, avec les premiers rapports venant de Zanzibar (1823,1870), du Burkina Faso (1925), d’Égypte (1887,1927), d’Afrique du Sud (1926-1927) et du Sénégal (1927-1928). Entre 1960 et 2010,20 épidémies confirmées en laboratoire se sont produites dans 15 pays africains, principalement en Afrique de l’Est [4]. La dengue est transmise par les moustiques *Aedes aegypti* et *Aedes albopictus*, originaires respectivement d’Afrique et d’Asie. Au Maroc, le vecteur *Ae. albopictus* a récemment été identifié dans la ville de Rabat [17]. Le Maroc est déclaré non endémique pour la dengue [69,96], mais l’infection par ce virus a été confirmée chez deux patients, un Marocain et un Ivoirien, tous deux provenant de Côte d’Ivoire, et qui se trouvaient à Abidjan durant l’épidémie de 2017 [10,47].

### Chikungunya (CHIKV)

Le CHIKV est un *Alphavirus* (famille des Togaviridae) transmis par les moustiques *Aedes.* Il représente une menace pour la santé mondiale. Le virus a été isolé pour la première fois en Tanzanie en 1953 et est désormais endémique dans de nombreux pays tropicaux d’Afrique et d’Asie, avec des taux de séroprévalence atteignant 75% [75,84]. Le virus était très probablement présent en Afrique avant 1952 et a été identifié à tort comme le virus de la dengue. La première mention possible du CHIKV en Afrique a été publiée au Caire en 1779 [26]. Un seul cas importé de chikungunya a été documenté au Maroc en 2017: une femme de 37 ans ayant contracté l’infection lors d’un séjour de 18 mois à Dhaka (Bangladesh). Compte tenu de la présence établie du vecteur *Aedes* sur le territoire marocain, ce cas soulève un risque potentiel de transmission locale du virus. Pour cette raison, le CHIKV doit être considéré dans le diagnostic différentiel des arthralgies chez tous les voyageurs revenant de pays où la transmission du virus a été démontrée [5,10].

### Zika (ZIKV)

Le ZIKV appartient au genre *Orthoflavivirus* (famille des Flaviviridae) [76]. Il est principalement transmis par *Ae. aegypti* [82] et *Ae. albopictus* [53,55]. Le ZIKV a été isolé pour la première fois en 1947 chez un singe macaque *(Macaca mulatta)* utilisé comme appât dans la forêt Zika en Ouganda, et en 1964 chez l’humain, toujours en Ouganda [30,31,85]. En 1969, le virus Zika (ZIKV) s’est propagé en Asie tropicale, atteignant des pays tels que l’Inde, l’Indonésie, la Malaisie et le Pakistan [40,50]. D’autres cas sont apparus en 2007 sur l’île de Yap, dans les États fédérés de Micronésie [54]. Le virus a également provoqué des flambées en Polynésie française en 2013 et 2014, entraînant environ 19 000 cas suspects [25]. Au Brésil, le premier cas de ZIKV a été signalé en 2013 et, en 2015, il s’est propagé aux États de Pernambuco, Rio Grande do Norte et Bahia dans la région du Nord-Est, suivis par d’autres zones dans les régions du Centre-Ouest et du Sud-Est [24,66]. En 2016, le ZIKV avait atteint la plupart des États du Brésil, à quelques exceptions près dans des zones reculées de l’Amazonie et dans les régions les plus méridionales. Le 1^er^ février 2016, l’Organisation mondiale de la santé (OMS) a déclaré que cette pandémie constituait une urgence de santé publique de portée internationale. Selon l’OMS, des cas de ZIKV ont été rapportés dans 86 pays [68], démontrant le fort potentiel de propagation de cet arbovirus combiné à l’expansion de ses vecteurs, notamment *Ae. (Stegomyia)) aegypti.* La maladie se propage de manière explosive, 16,6% des terres émergées de la planète (à l’exclusion de l’Antarctique) étant aujourd’hui menacées. Environ 6,22 milliards de personnes (79% de la population mondiale) vivent dans les zones à risque, dont la grande majorité en Asie du Sud, en Afrique tropicale, en Amérique du Sud, en Amérique du Nord et dans les pays du pourtour méditerranéen [98].

### Virus de la fièvre jaune (YFV)

Le virus YFV (famille des Flaviviridae, genre *Orthoflavivirus)* peut entraîner une hépatite hémorragique aiguë. La fièvre jaune est endémique dans les pays tropicaux et subtropicaux d’Afrique et d’Amérique du Sud [62], mais elle peut également survenir dans les régions tempérées [81]. Malgré l’existence d’un vaccin vivant atténué contre la fièvre jaune (17D) efficace, selon l’OMS environ 200 000 cas de fièvre jaune et 30 000 décès sont rapportés chaque année, avec près de 90% des cas et des décès survenant en Afrique [59]. Au Maroc, selon l’étude d’Amraoui en 2019, la transmission locale du YFV par *Ae. albopictus* récemment introduit au Maroc, est un scénario probable [5].

### Virus Usutu (USUV)

Le virus Usutu (USUV) est un virus transmis par les arthropodes (arbovirus) de la famille des Flaviviridae (genre *Orthoflavivirus)* qui appartient au complexe des virus de l’encéphalite japonaise. Il a été isolé pour la première fois en Afrique du Sud en 1959 [28]. L’USUV et le VNO partagent de nombreuses similitudes, notamment des relations phylogénétiques étroites, des écologies comparables et une tendance à la co-circulation dans la nature [94]. L’infection humaine par l’USUV est le plus souvent asymptomatique ou ne provoque que des signes cliniques bénins. Néanmoins, des cas neuro-invasifs d’USUV chez les humains, les animaux, particulièrement les oiseaux ont été signalés en Europe [93]. L’USUV a été détecté chez *Cx. pipiens* et *Cx. perexiguus* collectés en Algérie et dans le sud de l’Espagne [14]. L’USUV a été trouvé chez d’autres espèces de moustiques telles que *Cx. pipiens* [34], *Culiseta annulata, Ae. albopictus, Ae. japonicus* [94], *Ochlerotatus detritus, Oc. caspius* et *Anopheles maculipennis* s. l. collectés dans le nord de l’Italie [87,92]. Les deux virus ont été détectés chez des espèces locales de moustiques *Culex,* telles que *Cx. modestus* et *Cx. perexiguus,* dans les zones humides de certains pays d’Europe du Sud [51]. Une étude récente menée en Tunisie fait état de la première détection de l’USUV chez *Cx. perexiguus.* Elle a également montré qu’il existe une circulation importante du VNO et de l’USUV parmi les chevaux, ce qui est susceptible de provoquer des épidémies sporadiques à l’avenir [60].

En 2009, Figuerola a signalé que l’USUV circulait probablement au Maroc, des preuves sérologiques d’infection ayant été rapportées chez des oiseaux sauvages, bien qu’à un niveau plus faible que pour le VNO [43]. En 2012, l’exposition à l’USUV a été confirmée chez des chiens et des chevaux militaires au Maroc [32].

La plupart des vecteurs de l’USUV dans d’autres pays sont présents au Maroc selon Trari *et al.* [89], ce qui augmente le risque de l’émergence de cet arbovirus au Maroc.

La carte montre la distribution des arbovirus dans les différentes régions du Maroc depuis leur découverte et leur identification divisée en trois catégories: les foyers, les séropositifs et les importés.

### Facteurs influençant la diffusion future des maladies arbovirales transmises par les moustiques au Maroc

Le changement climatique pourrait avoir un impact direct sur la bio-écologie des arthropodes vecteurs, en favorisant la prolifération, l’émergence ou la disparition de certaines espèces qui peuvent être à l’origine de l’émergence de maladies vectorielles telles que la dengue, la fièvre de la vallée du Rift, le virus du Nil occidental, le chikungunya, etc. L’étude de l’écologie des vecteurs, de la génétique des populations, de leur sensibilité aux différentes familles d’insecticides et de leur compétence vectorielle, sont donc d’un intérêt capital pour comprendre l’épidémiologie de ces maladies et assurer leur contrôle.

## Conclusion

La présente revue fournit des informations sur la situation épidémiologique des arbovirus transmis par les moustiques et leurs vecteurs associés au Maroc.

Par ailleurs, et bien qu’aucun arbovirus majeur n’ait été identifié au Maroc (hormis les cas de FNO en 1996,2003 et 2010 dans la région du Gharb chez les chevaux), cette synthèse a mis en évidence un nombre important d’espèces marocaines impliquées dans la transmission.

Les espèces de moustiques de la sous-famille des Culicinae se distinguent en tant que vecteurs d’agents pathogènes, notamment *Aedes* (cinq espèces), *Culex* (six espèces) et *Culiseta* (une espèce). En septembre 2015, l’introduction d’Ae. *albopictus* à Rabat a été confirmée. Depuis lors, le risque d’émergence du CHIKV et du ZIKV avec des cas autochtones reste important, en particulier dans les villes densément peuplées du Maroc.

Des études entomologiques récentes au Maroc et dans les pays d’Afrique du Nord confirment la persistance de vecteurs ou de vecteurs potentiels et attestent que le risque d’épidémies est élevé. L’insuffisance des études, notamment en entomologie, est un autre facteur susceptible d’entraver la surveillance et la prévention des arboviroses.

## Contribution des auteurs et autrices

Samya JDI AA: prospection bibliographique, définition de la méthodologie et rédaction du manuscrit. Hanan HAZYOUN: rédaction du manuscrit. Moulay Anass LOUAH: correction et validation du manuscrit. Oumnia HIMMI: conception de l’étude, correction et validation du manuscrit.

## Conflit d’intérêt

Aucun conflit d’intérêts n’a été déclaré.
