# Supplementary material for: Risk of emergence of mosquito-borne arboviral diseases in Morocco: literature review
Source: Med Trop Sante Int. 2025 Aug 21;5(4):mtsi.v5i4.2025.671. [Article in French] doi: 10.48327/mtsi.v5i4.2025.671 (PMC12957995; doi:10.48327/mtsi.v5i4.2025.671)
Supplement: Related texts

# Research article: Risk of emergence of mosquito-borne arboviral diseases in Morocco: literature review

## Introduction

Mosquitoes are found worldwide and transmit several arboviruses (arthropod-borne viruses) to humans and animals [2]. The main viruses transmitted by mosquitoes belong to three families: Flaviviridae (West Nile virus, dengue virus, yellow fever virus), Togaviridae (e.g., chikungunya virus and O’nyong-nyong virus), and Phenuiviridae (e.g., Rift Valley fever virus) [1,2].

Each year, more than 700 million infections and nearly one million deaths worldwide are directly linked to vector-borne diseases [68,97]. These infections pose a major risk in Africa: 271 million people (23% of the population) are exposed to chikungunya, 750 million (63%) to dengue, 21 million (2%) to yellow fever and 406 million (34%) to the Zika virus. In all, 831 million Africans (70%) live in areas at risk from at least one of these diseases [95].

The West Nile virus (WNV), first reported in 1982, was first isolated in Morocco in 1996 and caused three equine epizootics [35]. In 2008, a serological survey of wild birds confirmed the presence of WNV in Morocco [43], and in 2012, the first human WNV infection was documented through serological evidence [37]. Recently, other arboviruses, such as the dengue and chikungunya viruses, have been identified in Moroccan patients who had traveled to Cote d’Ivoire and Bangladesh, respectively [10,11].

Due to its geographical location, climate, and exposure to the globalization of trade and travel (particularly with Europe and sub-Saharan Africa), Morocco is a high-risk area for the emergence or reemergence of pathogens (Fig. 1). This situation also favors the rapid spread of insect vectors, thereby exposing much of the population to these health threats [45,73].

**Figure 1:** Geographical distribution of arboviruses (imported cases, seropositive hosts, outbreaks) by region in Morocco since their appearance

The aim of this study is to identify the main arboviruses that pose a threat to human health in Morocco, as well as their epidemic vectors and the conditions that favor their emergence.

## Materials and methods

Relevant articles were searched for, selected, and included according to the PRISMA (Preferred Reporting Items for Systematic Reviews and Meta-Analyses) guidelines [61,72].

Studies published from the time the viruses were first identified in Morocco until now were searched for. They are based on systematic literature searches. Sources include the Culicidae of Morocco database [89], which traces the history of mosquitoes in the country from 1916 to 2017, as well as various search engines, such as Web of Science, Google Scholar, Scopus, ScienceDirect, PubMed, and the World Health Organization. The following keywords were used: “Morocco,” “mosquito-borne virus,” “mosquito-borne diseases,” “arbovirus,” “West Nile virus,” “Dengue virus,” “Rift Valley fever virus,” and “Chikungunya virus.” To further broaden the scope of the search, the terms “vector-borne disease genus,” “Africa,” and “emergence” were used.

To ensure the most relevant studies were included, we used a series of criteria (original research studies and studies referring to the genus of vector-borne diseases) for conference papers and journal articles on mosquito-borne arboviruses. Studies that did not meet the inclusion criteria (e.g., duplicates, lack of access to the full text, or a language other than French or English) were excluded. Figure 2 provides a detailed overview of the search process, highlighting the inclusion and exclusion criteria used in the publication selection methodology.

**Figure 2:** Identification of studies through databases and registers

## Results

A total of 668 articles were extracted from the databases. After applying the exclusion criteria, 208 articles were chosen for further analysis. Of those, 52 were excluded because they did not meet the inclusion criteria. Ultimately, 97 articles on the emergence of arboviral diseases transmitted by mosquito vectors were identified.

The PRISMA flowchart illustrates the methodology used to search for and select these studies [61,72].

### Historical overview of arboviruses and their current epidemiology in Morocco, with emphasis on their distribution and vectors

Arboviruses are a major global health problem and could become a significant public health issue in Morocco.

A total of 43 mosquito species have been reported in Morocco [91]. Fourteen of these species have been confirmed as potential vectors for transmitting pathogens to humans and/or animals [90]. Table I summarizes the main arboviruses and their vectors identified between 1996 and 2023.

**Table I:** Historical overview of arboviruses and their current epidemiological situation in Morocco, with emphasis on their distribution and vectors

Family	Genus	Disease name	Amplifying hosts	Year	History in Morocco	Specimen	Locations	Main vector	References
Phenuiviridae	*Phlebovirus*	Rift Valley fever (RVF)	Cattle, sheep, camels		In Morocco, RVF has not been recorded, but the virus is still present at the country’s borders with Mauritania			*Cx. pipiens, Ae. vexans*	7,21,44,52,56,89
Flaviviridae	*Orthoflavivirus,*	West Nile virus (WNV)	Birds	1996	First report on WNV	Horses, humans	The Atlantic coastal zone of northwest Morocco includes the following cities: Rabat, Kénitra, Khénifra, Benslimane and Sidi Slimane, as well as Casablanca	*Cx. pipiens, Ae. caspius, Ae. detritus, Ae. vexans, Ae. aegypti, Cx. modestus, Cx. mimeticus, Cx. perexiguus, Cx. theileri, Cx. impudicus, Cq. richiardii, Or. pulcripalpis*	6,8, 9,18,32,35,37,38,39,43,48,52,83,89
				2003	Re-emergence of WNV	Horses	Kenitra, Khenifra, Benslimane, Sidi Slimane, Casablanca		32,83
				2008	Circulation of WNV in Morocco	Wild birds	Province Sidi Allal Tazi Sidi Kacem		43
				2010	Re-emergence of WNV	Horses	Regions Kenitra, Khenifra, Benslimane, Sidi Slimane, Casablanca		32,37
				2012	A serological test	Humans (confirmed human cases), WNV in 11. 8% of 499 healthy individuals	Regions Meknès, Rabat et Kénitra		37
				2016	Seroprevalence	Horses and dogs	Agadir, Benslimane, Casablanca, Kenitra, Khenifra, Marrakech, Meknès, Salé, Sidi Slimane, Temara		32
Flaviviridae	*Orthoflavivirus*	West Nile virus (WNV)	Birds	2017	Seroprevalence	Horses, WNV in 31% of 840 horses	Atlantic plateaus of Gharb and pre-Rif region, North Atlas plains and plateaus, Atlas mountains and pre-Atlas region, Eastern plains and plateaus	*Cx. pipiens, Ae. caspius, Ae. detritus, Ae. vexans, Ae. aegypti, Cx. modestus, Cx. mimeticus, Cx. perexiguus, Cx. theileri, Cx. impudicus, Cq. richiardii, Or. pulcripalp*	16
				2020	Seroprevalence	Mosquitoes, horses	Mohammedia & Casablanca, Moulay, Tanger, Benslimane		9
				2021	Seroprevalence	Humans, Birds (wild birds?)	Casablanca		8
				2021	Seroprevalence	Horses	the small population of horses in this region)		48
		Usutu (USUV)	Birds as amplifying hosts	2016	Serological survey	Horses military dogs	Agadir, Benslimane, Casablanca, Kenitra, Khenifra, Marrakech, Meknès, Salé, Sidi Slimane, Temara	*Cx. pipiens, Ae. albopictus, Ae. caspius*	3,22,29,32,78
		Dengue (DENV)	Primates, humans	2018	Two cases of imported dengue fever in Morocco	Humans (two patients from Côte d’Ivoire, one Moroccan and one Ivorian who stayed in Abidjan during the 2017 epidemic)	At the Center for Virology, Infectious and Tropical Diseases (CVITD) at the Mohamed V Military Hospital in Rabat, Morocco	*Ae. albopictus Ae. aegypti*	10,11,17,64,89
Flaviviridae	*Orthoflavivirus*	Zika (ZIKV)	Primates, humans		No cases due to ZIKV have been reported in countries in the Western Mediterranean Region			*Ae. albopictus, Ae. aegypti*	41,49,52,67,79,90
		Yellow Fever (YFV)	Monkeys in sub-Saharan Africa, non-human primates as reservoir hosts		No cases of YFV in Morocco			*Ae. aegypti, Ae. albopictus*	6,33,71,80
Idem Togaviridae	*Alphavirus*	Chikungunya (CHIKV)	Primates, humans	2017	First case of Chikungunya imported into Morocco and North Africa	The patient returned to Morocco on August 15,2017, after staying in Dhaka, Bangladesh, for 18 months	At the Center for Virology, Infectious and Tropical Diseases (CVITD) at the Mohamed V Military Hospital in Rabat, Morocco	*Ae. albopictus, Ae. detritus, Ae. aegypti*	11,17,41,49,67,79,90

### West Nile virus (WNV)

WNV is a Flaviviridae virus *(Orthoflavivirus* genus) transmitted by several mosquito genera. WNV is a zoonotic arbovirus that is maintained in nature through an enzootic transmission cycle involving birds (mainly passerines) and orni-thophilic mosquitoes of the *Culex* genus. Birds contribute to the spread of the virus and act as amplifying hosts. WNV can also infect mammals, including humans. However, humans are considered “dead-end” hosts because they produce an insufficient level of viremia to infect mosquitoes and transmit the disease [20].

WNV infections in humans are usually asymptomatic (approximately 80%) [74]. Reported infections may present as a flu-like syndrome (West Nile fever), often accompanied by a rash, or more rarely, as a neuroinvasive syndrome with meningoencephalitis, flaccid paralysis, for which the case fatality rate is less than 10% (West Nile neuroinvasive disease) [23]. Approximately 1% of those infected develop severe symptoms [74]. WNV was first discovered in 1937 in the blood of a woman from the West Nile Province of Uganda who was suffering from a mild febrile illness [86]. Since then, sporadic cases and large outbreaks of West Nile fever have been reported in Africa, parts of Europe, the Middle East, Western Asia, Australia, and the Americas, with the first outbreak in North America occurring in 1999, extending to Central and South America afterwards [13,46,63]. Particularly large outbreaks have been documented in Greece, Palestine, Romania, Russia, France, and the United States [97].

According to official information from the National Veterinary Services in Morocco (Table II), WNV disease first appeared in 1996. An epizootic outbreak resulted in the deaths of 42 horses (out of 94 cases) and one human [43,88]. Subsequently, in 2003, the disease broke out in nine horses [83]. In 2010, the virus reappeared in the center and northwest regions of the country (Mohammedia, Casablanca, Benslimane, and Khemisset), resulting in eight deaths among 17 confirmed equine cases out of 111 suspected cases [16]. Since then, no clinical cases of the disease have been reported in the country. A total of seven seroepidemiological studies have been conducted in the northern region. One study was conducted in 11 of Morocco’s 12 regions, revealing the circulation of WNV in 4% of 346 native wild birds tested [43], 11.8% of 499 healthy human beings [37], 60% and 62% of 49 horses and 231 military dogs tested [32]. Three surveys with horses gave 31% positive out of 840 [16], 33.7% out of 92 [9], as well as 21.8% (255 out of 1 171) [48].

**Table II:** Seroprevalence of West Nile Virus (WNV) in Morocco

Geographical origin	Population studied	Samples	Positivity	Reference
Sidi Allai Tazi, Sidi Kacem Province	Wild birds	346	4%	43
Meknes, Rabat, Kenitra	Healthy people	499	11,8%	37
Kenitra, Khenifra, Benslimane, Sidi Slimane, Casablanca	Military horses	49	60%	32
	Military dogs	231	62%	32
Atlantic plateaus of the Gharb, Plains and plateaus of the Northern Atlas, Atlas Mountains and pre-Atlas region, plains and plateaus of the Oriental region	Horses	840	31%	16
Mohammedia, Moulay Bouselham, Tanger	Horses	92	33,7%	9
11 (out of 12) regions of the country	Horses	1 171	21,8%	48

Similarly, an entomological study confirmed the virus’s circulation among *Culex* mosquito populations in the country [9]. A second recent study on mosquito surveillance confirmed the presence of the virus in *Culex* populations in the country. Fifty-six additional mosquitoes (56 *Cx. pipiens pipiens)* from the Marrakech-Safi region tested positive for WNV. This is the first documented case of this virus circulating in *Cx. pipiens* mosquitoes in central Morocco [70].

### Rift Valley fever virus (RVFV)

RVFV is a zoonotic arbovirus that primarily affects cattle, sheep, goats, and camels, as well as humans. It is a member of the Phenuiviridae family, genus *Phlebovirus* [58], and was first identified in Kenya in 1931 [57]. It has caused numerous epidemics and epizootics in Africa, particularly in northwest Africa (Mauritania) and west Africa (Senegal). The circulation of RVFV remained limited to Africa until 2000, when the first cases outside of Africa were reported in Saudi Arabia and Yemen [58].

RVFV has never been reported in Morocco [7] However, the virus is still circulating along the border with Mauritania.

In 2010, Mauritania reported 30 human cases and 26 animal cases (in goats, sheep, and camels), suggesting the role of one-humped camels *(Camelus dromedarius)* in locally amplifying the virus for the first time [36,42]. In 2015 and again in 2020, 31 and 78 human cases of RVF were confirmed, respectively [12,19]. Seven mosquito species, belonging to three genera (*Culex* spp*.*, *Aedes* spp., *Mansonia* spp.), that are known to be vectors of RVFV have been detected in Mauritania [12].

The WHO has identified Morocco as being at risk of RVF emergence due to its shared border with Mauritania. Another report by the European Food Safety Authority in 2020 recognized the potential for the virus to spread from Mauritania to Morocco and then to Europe [65].

### Dengue (DENV)

Dengue is caused by the dengue virus (DENV), which belongs to the Flaviviridae family and the genus *Orthoflavivirus.* Epidemics have been documented in Africa since the 19 th century, with the first reports coming from Zanzibar (1823, 1870), Burkina Faso (1925), Egypt (1887, 1927), South Africa (1926-1927), and Senegal (1927-1928). Between 1960 and 2010, 20 laboratory-confirmed epidemics occurred in 15 African countries, primarily in East Africa [4]. Dengue is transmitted by the mosquitoes *Aedes aegypti* and *Aedes albopictus,* which are native to Africa and Asia, respectively. In Morocco, the *Ae. albopictus* vector was recently identified in the city of Rabat [17]. Although Morocco is considered non-endemic for dengue [69,96], infection with the virus has been confirmed in two patients: one Moroccan and one Ivorian from Cote d’Ivoire who were in Abidjan during the 2017 epidemic [10,47].

### Chikungunya (CHIKV)

CHIKV is an *Alphavirus* (family Togaviridae) transmitted by *Aedes* mosquitoes. It poses a threat to global health. First isolated in Tanzania in 1953, the virus is now endemic in many tropical countries in Africa and Asia, with seroprevalence rates reaching 75% [75,84]. It was most likely present in Africa before 1952, when it was mistakenly identified as dengue virus. The earliest known mention of CHIKV in Africa was published in Cairo in 1779 [26]. In 2017, Morocco documented a single imported case of chikungunya: a 37-year-old woman who contracted the infection during an 18-month stay in Dhaka, Bangladesh. Given the established presence of the *Aedes* vector in Morocco, this case raises the risk of local virus transmission. For this reason, CHIKV should be considered in the differential diagnosis of arthralgia in all travelers returning from countries where the virus is transmitted [5,10].

### Zika (ZIKV)

ZIKV belongs to the genus *Orthoflavivirus* in the Flaviviridae family [76]. It is primarily spread by *Ae. aegypti* [82] and *Ae. albopictus* [53,55]. ZIKV was first isolated in 1947 in a macaque monkey *(Macaca mulatta)* used as bait in the Zika Forest in Uganda, and in 1964 in humans, also in Uganda [30,31,85]. In 1969, ZIKV spread to tropical Asia, reaching countries such as India, Indonesia, Malaysia, and Pakistan [40,50]. Additional cases emerged in 2007 on Yap Island in the Federated States of Micronesia [54]. The virus caused outbreaks in French Polynesia in 2013 and 2014, resulting in approximately 19,000 suspected cases [25]. The first case of ZIKV in Brazil was reported in 2013. In 2015, it spread to the states of Pernambuco, Rio Grande do Norte, and Bahia in the Northeast region. Then, it spread to other areas in the Central-West and Southeast regions [24,66]. By 2016, ZIKV had reached most Brazilian states, with a few exceptions in remote Amazonian areas and the southernmost regions. On February 1, 2016, the World Health Organization (WHO) declared the Zika virus pandemic a public health emergency of international concern.

According to the WHO, ZIKV cases have been reported in 86 countries [68], demonstrating the high potential for this arbovirus to spread, particularly given the expansion of its vectors, notably *Ae. (Stegomyia) aegypti.* The disease is spreading explosively, and 16.6% of the world’s land area (excluding Antarctica) are now at risk. Approximately 6.22 billion people (79% of the world’s population) live in these areas, primarily in South Asia, tropical Africa, South America, North America, and countries bordering the Mediterranean [98].

### Yellow Fever Virus (YFV)

The YFV virus (Flaviviridae family, *Orthoflavivirus* genus) can cause acute hemorrhagic hepatitis. Yellow fever is endemic in tropical and subtropical countries to tropical and subtropical regions of Africa and South America [62], but it can also occur in temperate regions [81]. Despite the existence of an effective live attenuated vaccine against yellow fever (17D), the WHO reports approximately 200,000 cases and 30,000 deaths from YF each year, nearly 90% of which occur in Africa [59]. In Morocco, Amraoui’s 2019 study suggests that local transmission of YFV by *Ae. albopictus,* which was recently introduced to Morocco, is likely [5].

### Usutu virus (USUV)

The Usutu virus (USUV) is an arthropod-borne virus (arbovirus) belonging to the Flaviviridae family (genus *Orthoflavivirus)* belonging to the Japanese encephalitis virus complex. It was first isolated in South Africa in 1959 [28]. USUV and WNV share many similarities, including close phylogenetic relationships, comparable ecologies, and a tendency to co-circulate in nature [94]. Human infection with USUV is most often asymptomatic or causes only mild clinical signs. However, neuroinvasive cases of USUV in humans, animals, and especially birds have been reported in Europe [93]. USUV has been detected in *Cx. pipiens* and *Cx. perexiguus* collected in Algeria and southern Spain [14]. It has also been found in other mosquito species, including *Cx.pipiens* [34], *Culiseta annulata, Ae. albopictus, Ae. japonicus* [94], *Ochlerotatus detritus, Oc. caspius* and *Anopheles maculipennis* s.l., which were collected in northern Italy [87,92]. Both viruses have been detected in local *Culex* species, such as *Cx. modestus* and *Cx. perexiguus,* in wetlands in southern Europe [51]. A recent study conducted in Tunisia reported the first detection of USUV in *Cx.perexiguus.* The study also showed significant circulation of WNV and USUV among horses, which could cause sporadic epidemics in the future [60].

In 2009, Figuerola reported that USUV was probably circulating in Morocco. Serological evidence of infection was found in wild birds, though at a lower level than WNV [43]. In 2012, exposure to USUV was confirmed in military dogs and horses in Morocco [32].

According to Trari *et al.* [89], most USUV vectors in other countries are present in Morocco, increasing the risk of this arbovirus emerging there. The map shows the distribution of arboviruses in Morocco’s different regions since their discovery and identification. They are divided into three categories: outbreaks, seropositive, and imported.

### Factors Influencing the Future Spread of Mosquito-Borne Arboviral Diseases in Morocco

Climate change could directly impact the bioecology of arthropod vectors by promoting the proliferation, emergence, or disappearance of species responsible for vector-borne diseases, such as dengue fever, Rift Valley fever, West Nile virus, and chikungunya. Therefore, studying vector ecology, population genetics, sensitivity to different families of insecticides, and vector competence is paramount to understanding the epidemiology of these diseases and ensuring their control.

## Conclusion

This review summarizes the epidemiological situation of mosquito-borne arboviruses and their associated vectors in Morocco.

Although no major arboviruses have been identified in Morocco apart from WNF cases in the Gharb region in horses in 1996, 2003, and 2010, this summary highlights a significant number of Moroccan species involved in transmission. Mosquito species in the Culicinae subfamily are notable vectors of pathogens, including *Aedes* (five species), *Culex* (six species), and *Culiseta* (one species). In September 2015, *Ae. albopictus* was introduced in Rabat. Since then, the risk of CHIKV and ZIKV emerging with indigenous cases has remained high, particularly in Morocco’s densely populated cities.

Recent entomological studies in Morocco and other North African countries confirm the presence of vectors or potential vectors, indicating a high risk of epidemics. The lack of studies, particularly in entomology, may hinder surveillance and prevention efforts.

## Authors’ contributions

Samya JDIAA: literature review, definition of methodology, and manuscript writing. Hanan HAZYOUNE: Manuscript writing. Moulay Anass LOUAH: corrected and validated the manuscript. Oumnia HIMMI: study design and correction and validation of the manuscript.

## Conflict of interest

The authors declare no conflicts of interest.
